# COBRApy: COnstraints-Based Reconstruction and Analysis for Python

**DOI:** 10.1186/1752-0509-7-74

**Published:** 2013-08-08

**Authors:** Ali Ebrahim, Joshua A Lerman, Bernhard O Palsson, Daniel R Hyduke

**Affiliations:** 1 Department of Bioengineering, University of California, San Diego, 9500 Gilman Drive MC0412, La Jolla, 92093-0412, CA, USA; 2 Biological Engineering Department, Utah State University, 4105 Old Main Hill, Logan, UT 84322-4105, USA

**Keywords:** Genome-scale, Network reconstruction, Metabolism, Gene expression, Constraint-based modeling

## Abstract

**Background:**

COnstraint-Based Reconstruction and Analysis (COBRA) methods are widely used for genome-scale modeling of metabolic networks in both prokaryotes and eukaryotes. Due to the successes with metabolism, there is an increasing effort to apply COBRA methods to reconstruct and analyze integrated models of cellular processes. The COBRA Toolbox for MATLAB is a leading software package for genome-scale analysis of metabolism; however, it was not designed to elegantly capture the complexity inherent in integrated biological networks and lacks an integration framework for the multiomics data used in systems biology. The openCOBRA Project is a community effort to promote constraints-based research through the distribution of freely available software.

**Results:**

Here, we describe COBRA for Python (COBRApy), a Python package that provides support for basic COBRA methods. COBRApy is designed in an object-oriented fashion that facilitates the representation of the complex biological processes of metabolism and gene expression. COBRApy does not require MATLAB to function; however, it includes an interface to the COBRA Toolbox for MATLAB to facilitate use of legacy codes. For improved performance, COBRApy includes parallel processing support for computationally intensive processes.

**Conclusion:**

COBRApy is an object-oriented framework designed to meet the computational challenges associated with the next generation of stoichiometric constraint-based models and high-density omics data sets.

**Availability:**

http://opencobra.sourceforge.net/

## Background

Constraint based modeling approaches have been widely applied in the field of microbial metabolic engineering [1,2] and have been employed in the analysis [3-5] and, to a lesser extent, modeling of transcriptional [6-8] and signaling [9] networks. And, we’ve recently developed a method for integrated modeling of gene expression and metabolism on the genome scale [10].

The popularity of these approaches is due, in part, to the fact that they facilitate analysis of biological systems in the absence of a comprehensive set of parameters. Constraints-based approaches focus on employing data-driven physicochemical and biological constraints to enumerate the set of feasible phenotypic states of a reconstructed biological network in a given condition. These constraints include compartmentalization, mass conservation, molecular crowding [11], thermodynamic directionality [12], and transcription factor activity [13]. More recently, transcriptome data have been used to reduce the size of the set of computed feasible states [14-17]. Because constraints-based models are often underdetermined they may provide multiple mathematically-equivalent solutions to a specific question – these equivalent solutions must be assessed with experimental data for biological relevance [18].

We have previously published the COBRA Toolbox [19] for MATLAB to provide systems biology researchers with a high-level interface to a variety of methods for constraint-based modeling of genome-scale stoichiometric models of cellular biochemistry. The COBRA Toolbox is being increasingly recognized as a standard framework for constraint-based modeling of metabolism [20]. While the COBRA Toolbox has gained widespread use and become a powerful piece of software, it was not designed to cope with modeling complex biological processes outside of metabolism or for integrated analyses of omics data, and requires proprietary software to function. To drive COBRA research through this avalanche of omics and model increasingly complex biological processes [10], we have developed an object-oriented implementation of core COBRA Toolbox functions using the Python programming language. COBRA for Python (COBRApy) provides access to commonly used COBRA methods in a MATLAB-free fashion.

## Implementation

**Figure 1:**
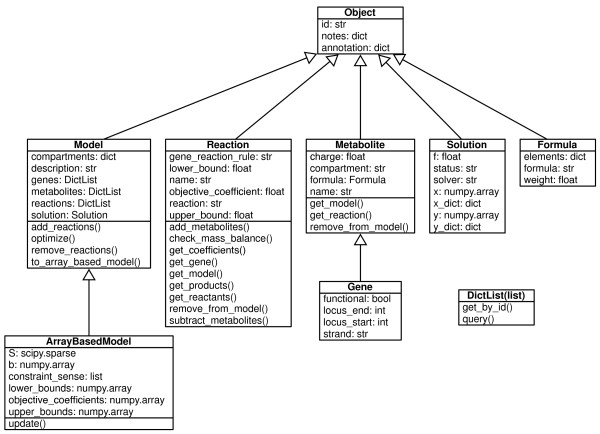
**Core classes in COBRA for Python with key attributes and methods listed.** Additional attributes and methods are described in the documentation.

The core capabilities of COBRApy are enabled by a set of classes (Figure 1) that represent organisms (Model), biochemical reactions (Reaction), and biomolecules (Metabolite and Gene). The core code is accessible through either Python or Jython (Python for Java). COBRApy contains: (1) cobra.io: an input/output package for reading / writing SBML [21] models and reading / writing COBRA Toolbox MATLAB structures. (2) cobra.flux_analysis: a package for performing common FBA operations, including gene deletion and flux variability analysis [18]. (3) cobra.topology: a package for performing structural analysis – the current version contains the reporter metabolites algorithm of Patil & Nielsen [22]. (4) cobra.test: a suite of unit tests and test data. (5) cobra.solvers: interfaces to linear optimization packages. And, (6) cobra.mlab: an interface to the COBRA Toolbox for MATLAB.

## Results and discussion

COBRApy is a software package for constraints-based modeling that is designed to accommodate the increasing complexity of biological processes represented with COBRA methods. Like the COBRA Toolbox, COBRApy provides core COBRA modeling capabilities in an extendible and accessible fashion. However, COBRApy employs an object oriented programming approach that is more amenable to representing increasingly complex models of biological networks. Moreover, COBRApy inherits numerous benefits from the Python language, and allows the integration of models with databases and other sources of high-throughput data. Additionally, COBRApy does not require commercial software for commonly used COBRA operations whereas the COBRA Toolbox depends on MATLAB. As the COBRA Toolbox is in wide use, it will likely be used as a development and analysis platform for years to come. To take advantage of legacy and future modules written for the COBRA Toolbox, COBRApy includes a module for directly interacting with the COBRA Toolbox (cobra.mlab) and support for reading and writing COBRA Toolbox MATLAB structures (cobra.io.mat).

In recent years, a number of software packages have been developed that employ stoichiometric constraint-based modeling approaches [23], such as Cell Net Analyzer [24], FASIMU [25], PySCeS-CBM [26], the Raven Toolbox [27], and the Systems Biology Research Tool [28]. While there is overlap in functionality between some of packages and COBRApy (Table 1), the other packages do not currently support the next generation models of metabolism and expression (ME-Models) [10] nor integration with the COBRA Toolbox for MATLAB. It is worth noting that the other software packages often contain a rich variety of functionality that is targeted towards other research topics, such as modeling signaling networks [24]. COBRApy continues the COBRA Toolbox’s tradition of providing an interactive / programmable framework for constraints-based modeling and is a new initiative of The openCOBRA Project [29]. Software downloads, tutorials, forums, and detailed documentation are available at http://opencobra.sourceforge.net.

**Table 1:** Features of available constraints-based programming packages

**Software package**	**GUI**	**FBA**	**FVA**	**M-models**	**ME-models**	**SBML**	**Strain design**	**Language**
**Cell net analyzer**	+	+	+	+		+	+	MATLAB
**COBRA toolbox**		+	+	+		+	+	MATLAB
**COBRApy**		+	+	+	+	+	*	Python
**fasimu**		+	+	+		+		bash
**PySCeS-CBM**		+	+	+		+		Python
**Raven**	+	+		+		+		MATLAB
**Systems biology research tool**		+	+	+				Java

+: feature is available; *: feature is accessible through the COBRA Toolbox.

### Core classes: model, metabolite, reaction, & gene

**Figure 2:**
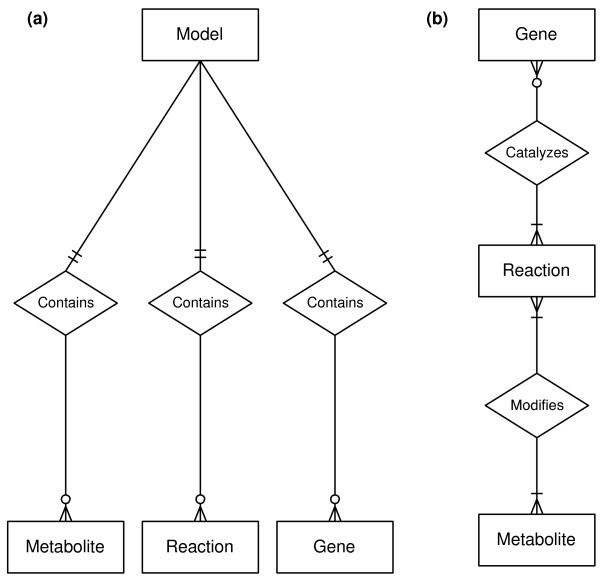
**Entity relationship diagrams for core classes in COBRApy. (a)** A Model contains Metabolites, Reactions, and Genes. **(b)** A Reaction may be catalyzed by 0 or more Genes. Reactions catalyzed by 0 Genes are spontaneous. A Reaction may be catalyzed by different sets of Genes. Reactions modify 1 or more Metabolites. A Reaction that modifies only 1 metabolite is an external boundary condition. A Metabolite may be modified by many different Reactions.

The core classes of COBRApy are Model, Metabolite, Reaction, and Gene. The Model class serves as a container for a set of chemical Reactions, including associated Metabolites and Gene products (Figure 2a). Within a Model, Metabolites are modified by one or more Reactions that may be spontaneous or catalyzed by one or more Genes (Figure 2b). The underlying genetic requirements for a Reaction to be active in a Model are supplied as a Boolean relationship [19], where each gene is referred to by a unique identifier. During the construction of a Model, the Model and the Reactions, Metabolites, and Genes are explicitly aware of each other. For example, given a Metabolite, it is possible to use the get_reaction() method to determine in which Reactions this Metabolite participates. Then the genes associated with these Reactions may be accessed by the Reaction.get_gene() method.

The object-based design of COBRApy provides the user with the ability to directly access attributes for each object (Figure 1), whereas with the COBRA Toolbox for MATLAB biological entities and their attributes are each contained within separate lists. For example, with COBRApy, a Metabolite object provides information about its chemical Formula and associated biochemical Reactions, whereas, with the COBRA Toolbox for MATLAB, one must query multiple tables to access these values and modify multiple tables to update these values.

### Key capabilities

COBRApy comes with variants of the published metabolic network models (M-Models) for *Salmonella enterica* Typhimurium LT2 [30] and Escherichia coli K-12 MG1655 [31]. These models can be loaded with the cobra.test.create_test_model function; with *S*. Typhimurium LT2 being the default model. Additionally, COBRApy can read SBML-formatted models [32] downloaded from a variety of sources, such as the Model SEED [33] and the BioModels database [34].

A common operation performed with M-Models is to optimize for the maximum flux through a specific reaction in a defined growth medium [35]. The *S*. Typhimurium LT2 model comes with a variety of media whose compositions are specified in the model’s media_compositions attribute. Here, we initialize the Model’s boundary conditions to mimic the minimal MgM medium [36] and then perform a linear optimization to calculate the maximal flux through the Reaction biomass_iRR1083_metals. Biomass_iRR1083_metals is a reaction that approximates the materials required to support *S*. Typhimurium LT2 growth in a minimal medium where approximately 0.3 grams dry weight *S*. Typhimurium LT2 are produced per hour. It is important to note that cellular composition can vary as a function of growth rate [37], therefore, for biological accuracy it may be necessary to construct a new biomass reaction if the simulated, or experimentally-observed, growth rate is substantially different [10,38].

Flux balance analysis of M-Models has enjoyed substantial success in qualitative analyses of gene essentiality [30]. These studies used simulations to identify which genes or synthetic lethal gene-pairs are essential for biomass production in a given condition. The lists of essential genes and synthetic lethal gene-pairs may then be targeted to inhibit microbial growth or excluded from manipulation when constructing designer strains [39]. COBRApy provides functions for automating single and double gene deletion studies in the cobra.flux_analysis module.

Because of the presence of equivalent alternative optima in constraint based-simulations of metabolism [18], many reactions may theoretically be able to carry a wide range of flux for a given simulation objective. Flux variability analysis (FVA) is often used to calculate the amount of flux a reaction can carry while still simulating the maximum flux through the objective function subject to a specified tolerance. Flux variability analyses can be used to identify problems in model structure [40] or ‘pinch-points’ in a metabolic network. COBRApy provides automated functions for FVA in the cobra.flux_analysis.variability module.

### Advanced capabilities

Because whole genome double deletion and FVA simulations can be time intensive with a single CPU, we have provided a function that uses Parallel Python [41] to split the simulation across multiple CPUs for multicore machines. Additionally, there are a wide range of legacy operations that are present in the COBRA Toolbox that can be accessed using mlabwrap [42]. MATLAB is only necessary for accessing codes written in the COBRA Toolbox for MATLAB; it is not necessary to run the majority of COBRApy functions.

## Conclusions

COBRApy is a constraint-based modeling package that is designed to accommodate the biological complexity of the next generation of COBRA models [10] and provides access to commonly used COBRA methods, such as flux balance analysis [35], flux variability analysis [18], and gene deletion analyses [43]. Through the mlabwrap module it is possible to use COBRApy to call many additional COBRA methods present in the COBRA Toolbox for MATLAB [19]. As part of The openCOBRA Project, COBRApy serves as an enabling framework for which the community can develop and contribute application specific modules.

## Availability and requirements

**Project name:** COBRApy version 0.2.1

**Project home page:**http://opencobra.sourceforge.net

**Operating systems:** Platform independent, including Java

**Programming language:** Python (≥2.6) / Jython (≥2.5)

Other requirements:

***Python*:** libSBML ≥ 5.5.0 [32]. Currently supported linear programming solvers: GLPK [44] through PyGLPK 0.3 [45], IBM ILOG/CPLEX Optimization Studio ≥ 12.4 (IBM Corporation, Armonk, New York), and Gurobi ≥ 5.0 (Gurobi Optimization, Inc., Houston, TX, USA).

• [Optional] Numpy ≥ 1.6.1 & Scipy ≥ 0.10.1 [46] for ArrayBasedModel, MoMA, and double_deletion analysis.

• [Optional] Parallel python [41] for parallel processing.

• [Optional] To directly interface with the COBRA Toolbox for MATLAB it is necessary to install mlabwrap [42], the COBRA Toolbox [29], and a version of MATLAB (Mathworks, Natick, Massachusetts, U.S.A.) that is compatible with the COBRA Toolbox.

***Jython*:** JSBML ≥ 0.8 [32,47].

•Currently supported linear programming solvers: GLPK for Java 1.0.22 [48], IBM ILOG/CPLEX Optimization Studio ≥ 12.4, and Gurobi ≥ 5.0.

•The COBRA Toolbox for MATLAB and ArrayBasedModel are not currently accessible from Jython.

**License:** GNU GPL version 3 or later.

## Abbreviations

COBRA: COnstraint-Based Reconstruction and Analysis; FBA: Flux balance analysis; FVA: Flux variability analysis; M-Model: Metabolic network model.

## Competing interests

This software was used by DRH, JAL, and BOP to develop the method that is the subject of a provisional patent application U.S. Provisional Application Serial No. 61/644,924 filed on May 9, 2012 entitled “Method for in silico modeling of gene product expression and metabolism”.

## Authors’ contributions

DRH conceived COBRA for Python. AE, JAL, and DRH contributed to various aspects of development and testing. All authors read and approved the final manuscript.
